# A Stochastic Burst Follows the Periodic Morning Peak in Individual *Drosophila* Locomotion

**DOI:** 10.1371/journal.pone.0140481

**Published:** 2015-11-03

**Authors:** Stanislav Lazopulo, Juan A. Lopez, Paul Levy, Sheyum Syed

**Affiliations:** Department of Physics, University of Miami, Coral Gables, Florida, United States of America; Alexander Fleming Biomedical Sciences Research Center, GREECE

## Abstract

Coupling between cyclically varying external light and an endogenous biochemical oscillator known as the circadian clock, modulates a rhythmic pattern with two prominent peaks in the locomotion of *Drosophila melanogaster*. A morning peak appears around the time lights turn on and an evening peak appears just before lights turn off. The close association between the peaks and the external 12:12 hour light/dark photoperiod means that respective morning and evening peaks of individual flies are well-synchronized in time and, consequently, feature prominently in population-averaged data. Here, we report on a brief but strong stochastic burst in fly activity that, in contrast to morning and evening peaks, is detectable only in single fly recordings. This burst was observed across 3 wild-type strains of *Drosophila melanogaster*. In a single fly recording, the burst is likely to appear once randomly within 0.5–5 hours after lights turn on, last for only 2–3 minutes and yet show 5 times greater activity compared to the maximum of morning peak with data binned in 3 minutes. Owing to its variable timing and short duration, the burst is virtually undetectable in population-averaged data. We use a locally-built illumination system to study the burst and find that its incidence in a population correlates with light intensity, with ~85% of control flies showing the behavior at 8000 lux (1942 μW/cm^2^). Consistent with that finding, several mutant flies with impaired vision show substantially reduced frequency of the burst. Additionally, we find that genetic ablation of the clock has insignificant effect on burst frequency. Together, these data suggest that the pronounced burst is likely generated by a light-activated circuit that is independent of the circadian clock.

## Introduction

The circadian clock is an intracellular molecular mechanism that regulates many biological processes including metabolism and behavior and is present in most organisms from bacteria to mammals, playing a significant role in their survival [1]. The fruit fly *Drosophila melanogaster* has historically been one of the most important model organisms for studies of the circadian clock. Fly locomotion studies in laboratory conditions have helped reveal the main components of the circadian clock as well as its primary zeitgebers, light and temperature. In a standard laboratory experiment, temperature is kept constant and the light pattern is varied in a rectangular cycle with a period of 24 hours—12 hours of constant light followed by 12 hours of complete darkness (12:12 LD). This pattern reveals the strong bimodal behavior of fruit flies with anticipated morning (M) and evening (E) activity peaks. Population averages of locomotor activity recordings produce two robust peaks at transitions between lights on/off states of illumination. To explain the M and E peaks, Pittendrigh and Daan proposed a dual oscillator model, with each oscillator separately controlling one of the peaks [2]. Later, molecular genetics identified ~150 neurons in the central nervous system as being primarily responsible for driving circadian rhythms of locomotor activity in the fly [3]. More recently, these neurons were classified into two distinct groups each responsible for one peak, supporting the Pittendrigh model [4–6]. The first group of pigment-dispersing factor (PDF) expressing lateral neurons (M cells) is responsible for the M peak while the second group of remaining neurons (E cells) is responsible for the E peak. The M cells are considered to be the master pacemaker that resets the E cells and drive the rhythms when zeitgebers are not present [7].

Natural light cycle is, however, different from the laboratory 12:12 LD pattern, and recent studies have shown that light pattern can affect clock function [8–11]. In natural light cycle, intensity is roughly sinusoidal during the day and remains constant at ≲ 1 lux at night. The spectrum of sunlight is also very different from that of the artificial light that is used in standard experiments. Sunlight spectrum is distributed uniformly over a wide range of wavelengths, including ultraviolet and infrared. In contrast, luminescent spectrum has a few narrow peaks and incandescent spectrum is shifted to the infrared range. Recent studies with a more naturalistic light sources and patterns have shown that M and E cells can take over each other’s functions in certain conditions. Under dim light with M cells inactive, E cells can apparently generate the M peak [9], while strong light intensity and temperature can force M cells to generate the E peak [10]. These and other data led to the proposal that instead of two, there are many oscillators which interact to produce circadian patterns of locomotor activity [12]. Moreover, experiments under natural and semi-natural conditions reveal an additional activity peak (A peak) in the middle of the day [13–16]. These results show inconsistencies with the simple dual oscillator model and the standard 12:12 LD experimental paradigm in explaining features of fly locomotor activity.

In addition, analysis of fly locomotion under LD conditions is frequently done on population-averaged data. While such averaging is effective in revealing features like the M and E peaks that appear in close synchrony among individuals, it is less effective in identifying other potentially important locomotion features that lack temporal synchrony. However, studying such locomotor features of single fly recordings can circumvent this limitation in current methods of analysis.

Here we describe a transient burst of activity that appears only in single fly activity data on average ~2.3 hours after the start of daylight in wild-type and clock mutant flies. The burst stands out from other peaks in single fly data due to its extremely high magnitude of ~5 times that of the M peak (consolidated in 3 minute bins), and its appearance in ~85% of flies, despite being temporally stochastic. We used a custom-built light control system installed in a typical fruit fly incubator to investigate the burst. The system consists of white LED light sources, light sensors, and integrated hardware-software, which allow one to generate and measure light patterns of any shape with intensity up to 8800 lux with 5% precision range. The apparatus allows us to go beyond the limits imposed by the standard 12:12 LD experiments for comprehensive analysis of fly locomotion.

## Materials and Methods

### Fly strains and recording

The following fly strains were used in this study: yellow-white (*yw*), *iso31* [17], 2U (2202u), *per* ^*0*^ [18], *dbt* ^*AR*^ [19], *cry* ^*01*^ [20], *norpA* ^*36*^ (or *norpA* ^*P24*^ [21]), *ninaE* ^*17*^[22,23] and *hdc* (histidine decarboxylase w^1118^; Mi{ET1}Hdc^MB07212^ [24]). The 2U strain is a w^1118^ (isoCJ1), a Canton-S derivative [25]. w^1118^; Mi{ET1}Hdc^MB07212^ is a mutant with transposon insertion in hdc, that leads to deficiency in histamine synthesis [26]. All flies were raised on *Drosophila* medium (corn meal, agar, molasses, and yeast). Only male flies of age 2–7 days were used in the experiments.

In all experiments, locomotion was recorded in 20 seconds bins using activity monitors (DAM 5, TriKinetics Inc., MA). Flies were placed in individual glass tubes in a monitor with an infra-red (IR) beam bisecting each tube perpendicular to its long axis. Every time a fly intersects the beam, activity is detected by the IR sensor and counted into the time series. Monitors were placed in an environmental chamber (Percival Scientific, IA) maintained at 25°C and 70–80% relative humidity. Light was produced by our control electronics as described below.

### Data analysis

Data from the monitors were visualized and processed with custom written Matlab (MathWorks Inc., MA) scripts. Data were binned in 3 minutes for single fly analysis and in 30 minutes for population- level analysis. The Lomb-Scargle (LS) method was used to calculate power spectrum of fly locomotion [27,28]. The method has several advantages over the more commonly used fast Fourier transform algorithms in that LS can be used with unequally spaced data, produces a higher resolution power spectrum and allows for the calculation of the statistical significance of peaks in power spectrum [29]. In this work, we utilized a publicly available implementation of LS (http://www.mathworks.com/matlabcentral/fileexchange/22215-lomb-normalized-periodogram).

### Burst detection

The burst was detected in activity data binned in 3 minutes. Within one light cycle, the burst was identified as the bin with the highest activity count. Occasionally, multiple bins with comparably high counts appeared within ~10 min window. In such cases, an average bin was calculated without significant error to burst count or timing. Finally, if the bin with maximum activity occurred within ± 5 min of light transition (< 5%), then it was considered part of the fly’s startle response and not the burst in activity discussed here. These criteria resulted in the burst having a count ≳10 standard deviations above mean activity. For example, burst count ≳30 per 3 minutes for *yw* flies, while the average activity count in that population is 2.84±2.38 per 3 minutes (mean ±standard deviation). The three minutes binning was selected as optimal as this maximizes counts/bin for our data. Resizing bins smoothes out the data making it difficult to detect sharp increases in activity.

### Illumination system

**Fig 1:**
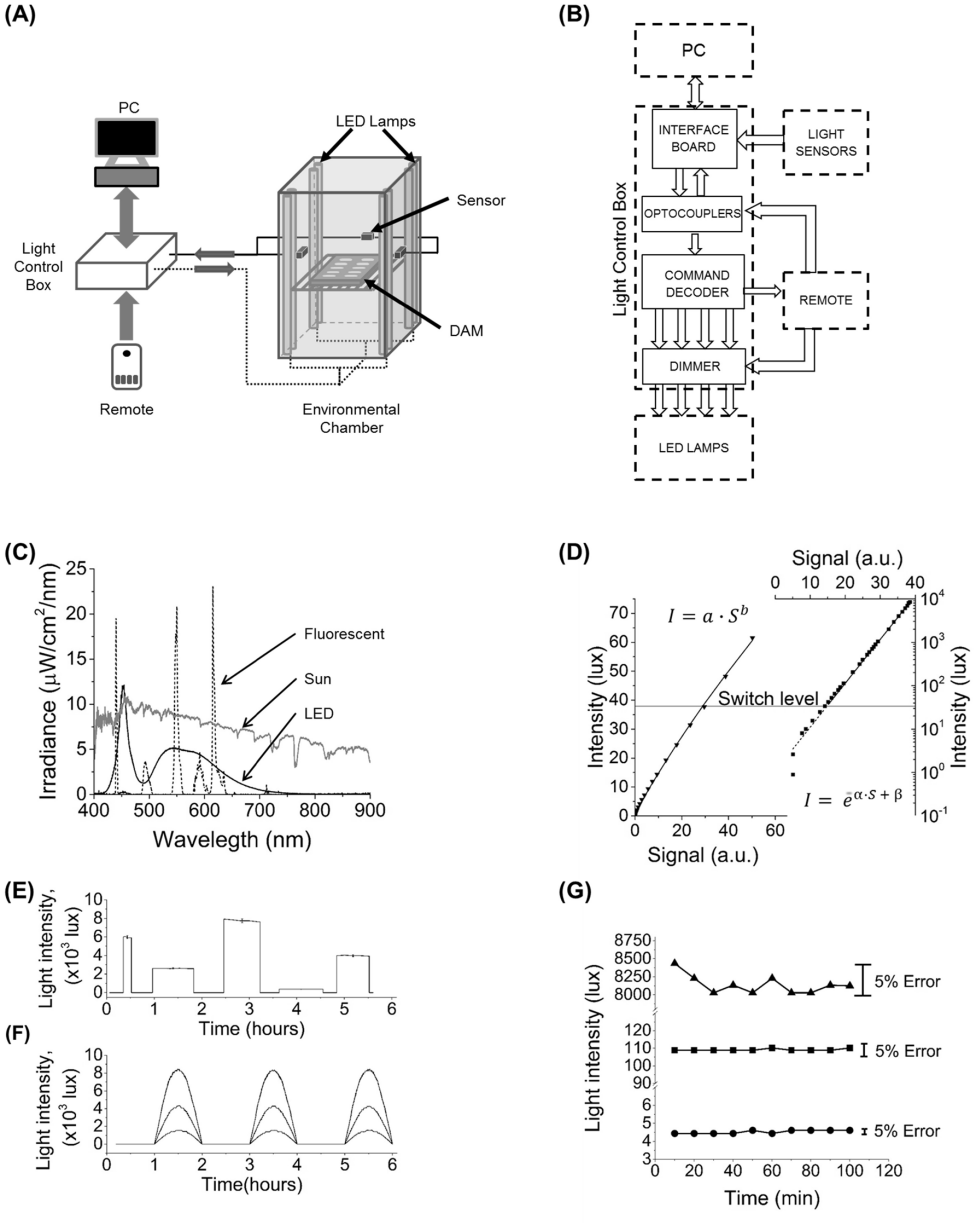
Illumination system and its characteristic. (A) Schematic of the experimental setup and the production and detection of light. Each of the eight LED lamps are controlled from a computer to provide illumination between 0 and 8800 lux. The level of illumination is reported to a computer by three pairs of photodiode-based sensors. (B) Information flow between main components of light control box and peripheral devices. Interface board receives control signals from computer and forwards them to the command decoder which interprets the signal to set the desired light intensity. (C) Typical irradiance spectra for the installed LED lamp (solid line) compared to the previous fluorescent lamp (dashed line) and local (25° 43' 24.26'' N, 80° 16' 45.89'' W) sunlight (grey line, not to scale). LED lamp spectrum is broadly distributed, while fluorescent irradiance is concentrated in three narrow peaks. The LED peak at 450 nm coincides with one of the peaks in the action spectrum of *Drosophila* CRYPTOCHROME. Compared to fluorescent lamps, the LED spectrum better approximates light conditions in the natural environment. (D) Calibration data for a low range (left) and a high range (right) sensor. Data shown in symbols and fits in solid lines. The low range sensor has a linear response while the high range sensor has a logarithmic response. For the sensor shown parameters were a = 0.29,b = 0.86,α = 2.33x10^-2^, β = 6.71x10^-2^. Software controlling the light system automatically switches from low to high range sensor at 38 lux (horizontal line). (E) Square patterns of different LED intensities and durations. Error bars at peak values represent 5% variation with respect to each target value. (F) Half-sine light cycles of different maximum intensities. The half-sine shape with a period of 24 hours closely simulates variations in daily sunlight intensity. (G) Stability of the system for three different LED intensities each maintained for 100 minutes. For all tests, intensity variations were limited to 5% error bounds.

Specific light conditions for behavioral experiments were created with our locally-built light control system. The system consists of lamps and light sensors installed inside the incubator (Percival Scientific, IA) and communicating with an external light control box and a computer or manual remote control (Fig 1A and 1B). Electronics gathered in the light control box include an interface board (Part#1018_2, PhidgetInterfaceKit, Phidget Inc., Canada), optocouplers, a command decoder, and a dimmer (Fig 1B and S1 Fig). The light control box decodes commands from the host, distributes them to each lamp and also sends data from the light sensors to the host. The dimmer consists of four identical parts, one for each group of lamps. Its function is to dim the light by varying the input voltage. The input voltage is determined by a digital potentiometer which receives signal from the command module to change by multiples of 1/100 of contiguous resistor values. This varies the input voltage and gives precise control of lighting from 0–100% of maximum light intensity. The manual remote control can be used in the event communication with the computer is lost (Fig 1 and S1 Fig). For more design details see Supplementary Materials.

Light-emitting diodes (LED) were chosen over fluorescent lamps as the light source for several reasons. A large part of fluorescent lamp irradiance is in the range of 600–650 nm (red color) (Fig 1C), where fly vision is weakly sensitive, and CRYPTOCHROME (CRY) absorbance is effectively zero. Fluorescent lamps also have a relatively short shelf life and gradually lose brightness and color temperature. Critically, these lamps cannot be dimmed to arbitrarily small values because they have a minimum power requirement for producing a discharge. In contrast, the white LED light source allows smooth control in its entire range of irradiation from 0 to 100%. In addition, its spectrum is blue shifted relative to fluorescent sources, which makes it closer to sun light (Fig 1C). In our system, we use LED lamps (LED-88020-120V Neptun Light Inc., IL) with total number of 576 LEDs per lamp housed in a 4 ft. aluminum (T8 standard) support with a sturdy polycarbonate frosted cover.

For real-time monitoring of the light levels between 0 and 8800 lux (for LEDs, 1 lux = 0.243 μW/cm^2^), we used two types of sensors. The first type (Part#1143_0, Phidget Inc., Canada) has a logarithmic response to light covering up to 70 000 lux but becomes unreliable at intensities below 10 lux (Fig 1D). To cover these low light intensity levels a second type of sensor (Part#1142_0, Phidget Inc., Canada) sensitive to light between 1 and 1000 lux was installed (Fig 1D). Sensors were modified with diffusors to reduce their sensitivity to the direction of the incoming light and each was individually calibrated with a light meter (LM-200LED Amprobe, WA). Three groups of two (one of each type) sensors were installed on the incubator walls (Fig 1A).

### Software

A graphical user interface was developed using Matlab. The program allows setting up rectangular (instantaneous lights on/off), triangular (linear increase/decrease) and half wave rectified sine (half period sine/half period lights-off) light patterns. For each type of pattern, the user can choose the length of day and night, the maximum intensity and the length of the experiment. If the user requires a special pattern that is not built in, the custom option is also available. The software is available upon request.

## Results

### A flexible illumination system that better mimics natural light conditions

The illumination system was installed in a standard biological incubator (Fig 1A) to substitute previous built-in fluorescent lighting in order to improve the quality of the experiments. The control box (Fig 1B) permits the production of arbitrarily-shaped light patterns with the 8 LED lamps that provide spectrum and intensity close to those of natural light. The LED lamps used in the system have a spectrum consisting of high peak at 450 nm that coincides with one of the CRY absorbance peaks and a broad peak in the green to red region (Fig 1C). The fluorescent light spectrum has sharp peaks at 440 (blue color), 550 (green color) and 615 (red color) nm, while, in other regions, the spectrum shows smaller peaks or no irradiance at all (Fig 1C). The *Drosophila* CRY action spectrum has two peaks, one from pterin and another from flavin absorbance with wavelengths of 380 and 450 nm, respectively [30]. Calculation of the power per unit area from the 450 nm peak in the LED spectrum and the 440 nm peak in the fluorescent spectrum gives 300 μW/cm^2^ for the former and 49 μW/cm^2^ for the latter, at the same light level of 4000 lux. This makes the LEDs ~6 times stronger than fluorescent lamps as an entraining tool. Additionally, a smoother power distribution of their spectrum makes LED lamps a better approximation than fluorescent lamps to sunlight (Fig 1C).

The light sensors are analog photo resistors which need calibration to specific ambient light. Calibration of sensors was performed with a light meter with 5% uncertainty. For low range sensors, the calibration curve closely follows the function *I* = *ɑ∙S* ^*b*^ *(I*–light intensity, *S*–analog signal, *ɑ* and *b*–fit parameters) for data points from 0 to 65 lux (Fig 1D). For high range sensors, the data points follow the function *I* = e^α∙S+β^(α and β –fit parameters) deviating from this only below 10 lux (Fig 1D). To obtain maximum accuracy, 38 lux was chosen as the light level at which the acquisition software automatically switches from one type of sensor to the other.

The light system can produce illumination of any arbitrary shape with uncertainty of less than ±2.5%. For example, a user can run a standard square pattern with different intensities and day and night durations (Fig 1E). For simulating shape of natural light cycle, one can use a half wave rectified sine pattern (Fig 1F) or a simpler trapezoidal pattern with linear change of light at “dawn” and “dusk”. The light system also shows high stability with fluctuations within ±2.5% of the target level (Fig 1G).

### Population-level studies reveal differential light response of M and E peaks

Control *yw*, *iso31* and 2U flies were first entrained for three days in standard 12:12 LD conditions and placed in constant darkness for two weeks. Power spectra of the locomotor data were calculated using the Lomb-Scargle method (S2 Fig bottom panels). Although in LD all three control strains show similar activity with strong M and E peaks (S2A Fig), in constant darkness (DD) their behaviors diverge. In DD, the M and E peaks of both *iso31* and *yw* flies gradually dissipate causing significant loss of circadian rhythmicity by the 5th day after the start of constant conditions (S2B and S2C Fig). However, in 2U flies only the M peak dissipates while the E peak shifts to the middle of the day, maintaining a strong circadian rhythm, remarkably, for more than 2 weeks of recording (S2D Fig).

**Fig 2:**
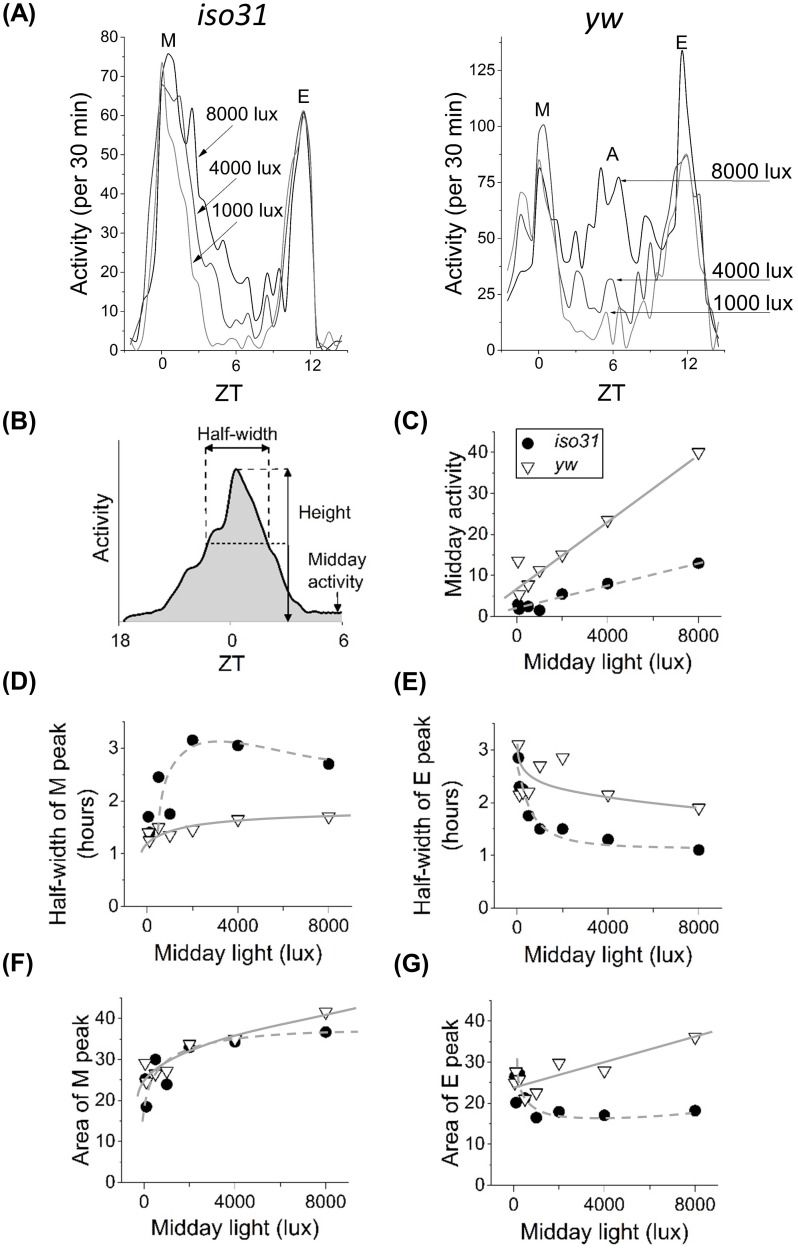
Locomotor activity at different light intensities. Population-averaged activity profiles of *iso31* (panel A, left) and *yw* (panel A, right) for different midday light levels. Only *yw* shows appearance of the A-peak for ~8000 lux. (B) Cartoon of the peak properties plotted in C-G. *iso31* flies shown in black circles and *yw* flies in white triangles, grey solid and dashed curves are guides to the eye. The midday activity (C) is an average activity of 4 hours from 4 to 8 hours after lights-on. It increases with light intensity for both fly strains. (D) Half-width of the M peak increases while (E) half-width of the E peak decreases with light intensity. Areas of M and E peaks (F and G) represent total activity from 3 hours before to 3 hours after respective peak. With the exception of the E-peak of *iso31*, areas under the peaks increase with increase in light intensity. Legend in (C) applies to panels C-G.

To further investigate differences in the locomotor behavior of control strains, we next studied *yw* and *iso31* flies using trapezoidal light cycle, which features a linear change in light levels around dawn and dusk. The ramp rate between zero and the maximum was varied depending on the maximum lux target such that the increase/decrease always took one hour. For both strains, population-averaged analyses show presence of M and E peaks for all days (Fig 2A). In *iso31*, high light levels produce extended morning activity, resulting in flies being active throughout the middle of the day. In contrast, in *yw* flies high light levels instead produce the ‘A’ peak around ZT 6 (Fig 2A). Our observation that the A peak appears with increasing illumination is consistent with results reported by De et. al. [15] and at odds with that of Vanin et. al. who attributed appearance of the peak to temperature oscillations in the environment [13]. Although different in terms of the A peak, both *iso31* and *yw* flies show increase in midday activity with increase in light levels (Fig 2C). The response rates are, however, different and likely related to the midday A peak being present only in *yw* flies (Fig 2C solid and dashed lines). The two strains also show differences in how their M and E activity peaks respond to changes in light. The data show that while both the half-width and the area of the M peak increase with illumination (Fig 2D and 2F), half-width of the E peak paradoxically decreases with higher light intensities (Fig 2E). The monotonic decrease in half-width of the E peak with increasing light intensity (Fig 2E) maybe reminiscent of the previously reported light-sensitive contribution of DN1 cells to the evening peak [10]. Because of this narrowing of the E peak, its area also decreases in *iso31*, but not in *yw* (Fig 2G). These population-averaged data reveal significant differences in light response by the M and E activity peaks. The differences between the peaks may reflect differences in sensitivity of the underlying M and E cells that generate activity. Additionally, the data show dissimilarities in locomotor patterns between two commonly used fly strains, underscoring the importance of genetic background in behavior [31].

### Single fly analyses uncover strong burst in activity

**Fig 3:**
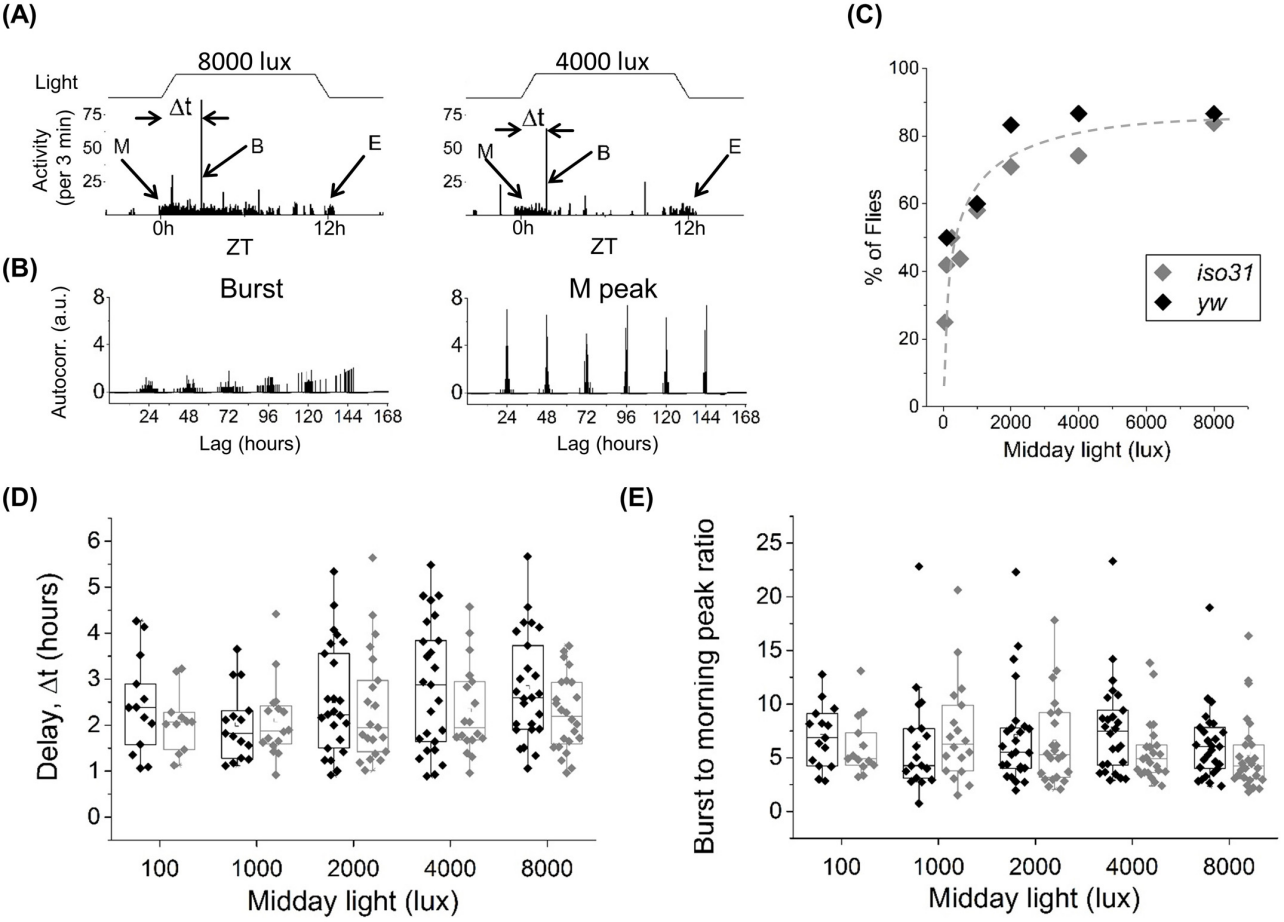
Individual flies show significant burst of activity after start of morning activity. (A) Examples of activity (3 minutes bins) for two different flies with midday light intensity of 8000 (left) and 4000 (right) lux. Morning/evening peaks denoted as “M” and “E” respectively, burst of activity as “B”, delay of B peak after lights turns on as Δt (activity in black columns and light patterns in black line on top). (B) Normalized autocorrelation functions for the stochastic burst (left) and the periodic M peak (right) for a fly measured in LD for 6 days. (C) Number of flies (in percent of total number), that showed the burst at different midday light intensities. Percentage of flies showing burst increases monotonically with light intensity. Grey dashed curve is a guide to the eye and not a fit to the data. (D) Onset of the burst after lights-on for *iso31* and *yw* for different midday light intensities. Δt≈110−150 minutes on average, independent of light intensity and genetic background. (E) Ratio of the burst activity to the average morning activity for *iso31* and *yw* at different midday light levels. Average value of the ratio ~5 for *iso31* and ~6 for *yw*. Legend and number of flies, *iso31* (N = 32) and *yw* (N = 32), in (C) applies to panels C-E.

Population-averaged recordings are ideally suited for examination of locomotor features that, like the M and E peaks, are synchronized among individuals subjected to the same zeitgeber cycle. We wondered, however, if there are important features that are unsynchronized in the population and so, are typically missed in population level activity analyses. Our examination of individual flies shows the presence of an unexpected burst of activity ~10 standard deviations larger than average locomotor activity, yet of short duration, lasting ~2–3 minutes (Fig 3A and S3 Fig). Although the burst is the most prominent feature in single fly recording (Fig 3A and S4A Fig), it appears stochastically after lights turn on resulting in weak autocorrelation compared to the M peak which is tightly coupled to the zeitgeber (Fig 3B). Its temporal stochasticity and transience imply that either averaging over multiple flies or binning in longer time bouts leads to masking of the burst by the M peak (S5 Fig). The activity burst is detected in *yw*, *iso31*and 2U fly strains with roughly equal frequency. Since all three control flies show similar burst statistics, from here on additional analyses will focus on *yw and iso31* strains. The frequency of the burst increases with daytime light intensity, with up to ~85% of flies (N = 32 for each strain) showing the burst at ~8000 lux illumination (Fig 3C). Consistent with this strong correlation with light intensity, the burst is rarely observed during nighttime in LD experiments and not observed during the subjective day in DD conditions (data not shown). Further analysis shows that regardless of light intensity, the activity burst appears in the 0.5–5 hours range after the start of the day in both *yw* and *iso31* flies, with an average delay Δt ~ 2.3 hours (Fig 3D). Additionally, the magnitude of the burst is on average ~5 times larger than the M peak activity and, similar to Δt, independent of light intensity or genetic background (Fig 3E). Importantly, unlike the M or E peak which vary significantly between control fly lines (Fig 2C–2G), the frequency, timing and relative magnitude of the burst appear largely independent of background, suggesting that the burst behavior is likely a fundamental mode of activity in flies.

We find that the shape of the zeitgeber cycle (square, trapezoid, sine) does not dramatically alter burst characteristics. Choice of the illumination source (LED, fluorescent) does, however, affect burst frequency in the fly population. We find that for the same lux level, LEDs evoke burst in 15% more flies on average (data not shown), suggesting that spectral differences between the two sources (Fig 1C) are important in eliciting the behavior. Finally, to test if the center of the tube is critical to detection of the burst, we shifted the IR beam position closer to the food or the cotton end and found no major alterations in the burst characteristics (data not shown).

### The burst requires light but not circadian clock

**Fig 4:**
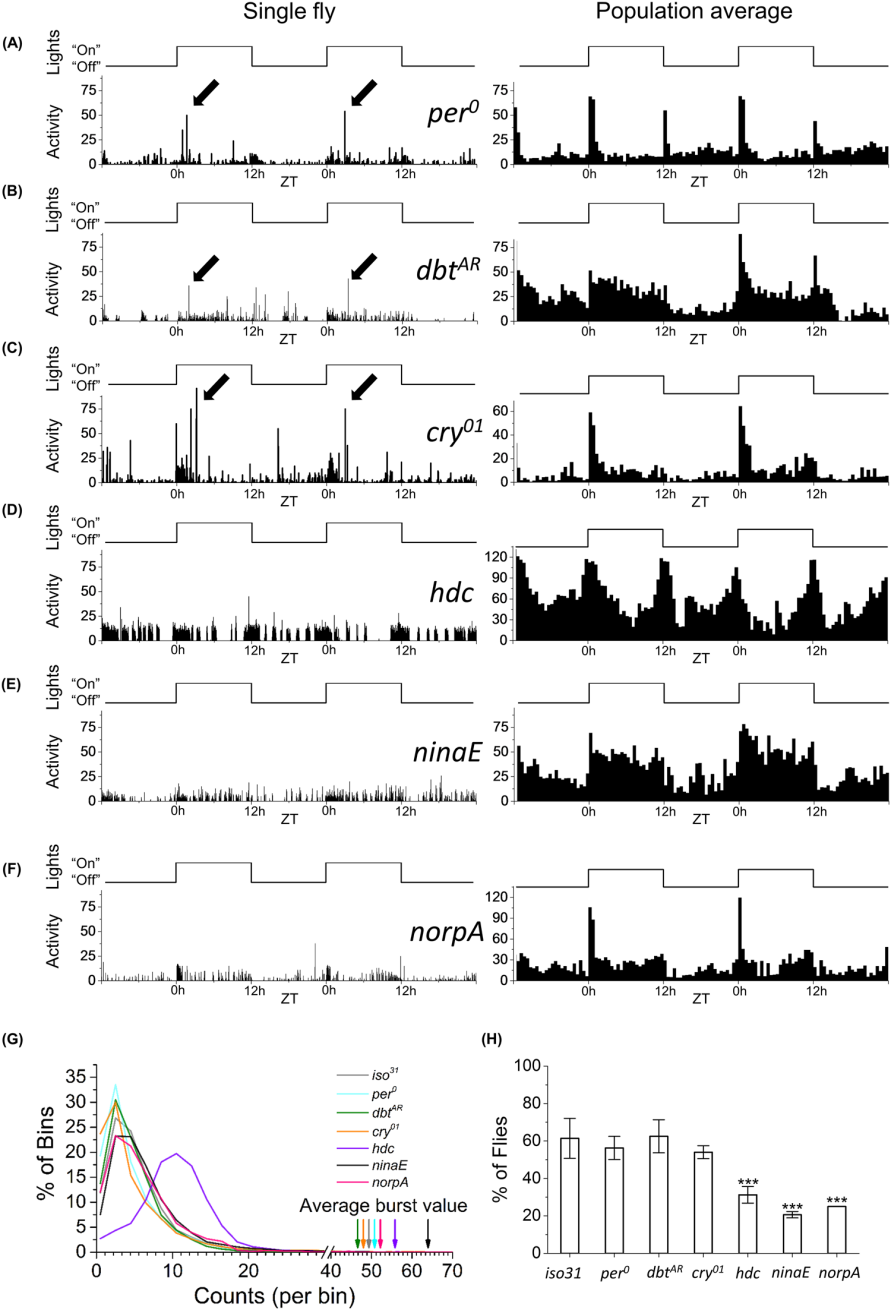
Light/dark experiments with *Drosophila* mutants suggest light is required for activating burst in locomotion. (A) *per* ^*0*^, (B) *dbt* ^*AR*^, (C) *cry* ^*01*^, (D) *hdc*, (E) *ninaE*, and (F) *norpA* under 12:12 LD conditions. Single fly (left, 3 minutes bins) and population averaged (right, 30 minutes bins). (A-C) Black arrows indicate bursts. (G) Probability distribution of the 3 minutes bins in activity. Grey line–*iso31* (N = 25, for 5 days), cyan–*per* ^*0*^ (N = 14, for 5 days), green line—*dbt* ^*AR*^ (N = 16, for 2 days), orange line–*cry* ^*01*^ (N = 32, for 3 days), violet line–*hdc* (N = 16, for 5 days), black line–*ninaE* (N = 10, for 5 days), and pink line–*norpA* (N = 8, for 2 days). Corresponding arrows show average value of activity burst in each population. (H) Percent of flies per day that showed burst under 12:12LD. Stars denote significant difference compared to *iso31* control flies (p<0.05, Mann-Whitney-Wilcoxon test). For *norpA*, same number of flies showed bursts each day, providing insufficient statistics for error bars.

Since a 12:12 LD light cycle also drives the circadian clock near its natural period, our experiments raised the possibility that the endogenous clock may play a role in timing the activity burst. However, we find that similar to control strains, *per* ^*0*^, *dbt* ^*AR*^ and *cry* ^*01*^ mutants show the burst within the usual ~0.5–5 hours after lights turn on (Fig 4A–4C, left panel, arrows). Comparable to that in *iso31* control, clock mutants also show ~10–12 times more activity within the burst relative to basal levels (Fig 4G, arrow and maximum in distribution) and without a significant difference in burst frequency (Fig 4H). These data together suggest that a functional circadian clock is not required for the generation of the burst in activity. In contrast, partially blind mutant *ninaE* ^*17*^ that lacks rhodopsin Rh1 and completely blind mutant *norpA* ^*36*^ show significantly lower frequencies of the burst (Fig 4H). *hdc* flies, which have impaired light transduction owing to depleted histamine levels, also show reduced frequency of bursts (Fig 4D and 4H) despite showing increased levels of basal activity compared to other strains (Fig 4D, right-shifted maximum in violet curve). The suppression of burst in the *hdc* animals coexisting with increased locomotion suggests the burst has weak correlation to activity. Interestingly, *hdc* flies show wildtype M and E peaks in LD cycles, implying that sufficient light was available to drive their M and E cells but not those responsible for the burst (Fig 4D, right panel). These results suggest that light is a major driver of the observed burst in fly activity.

**Fig 5:**
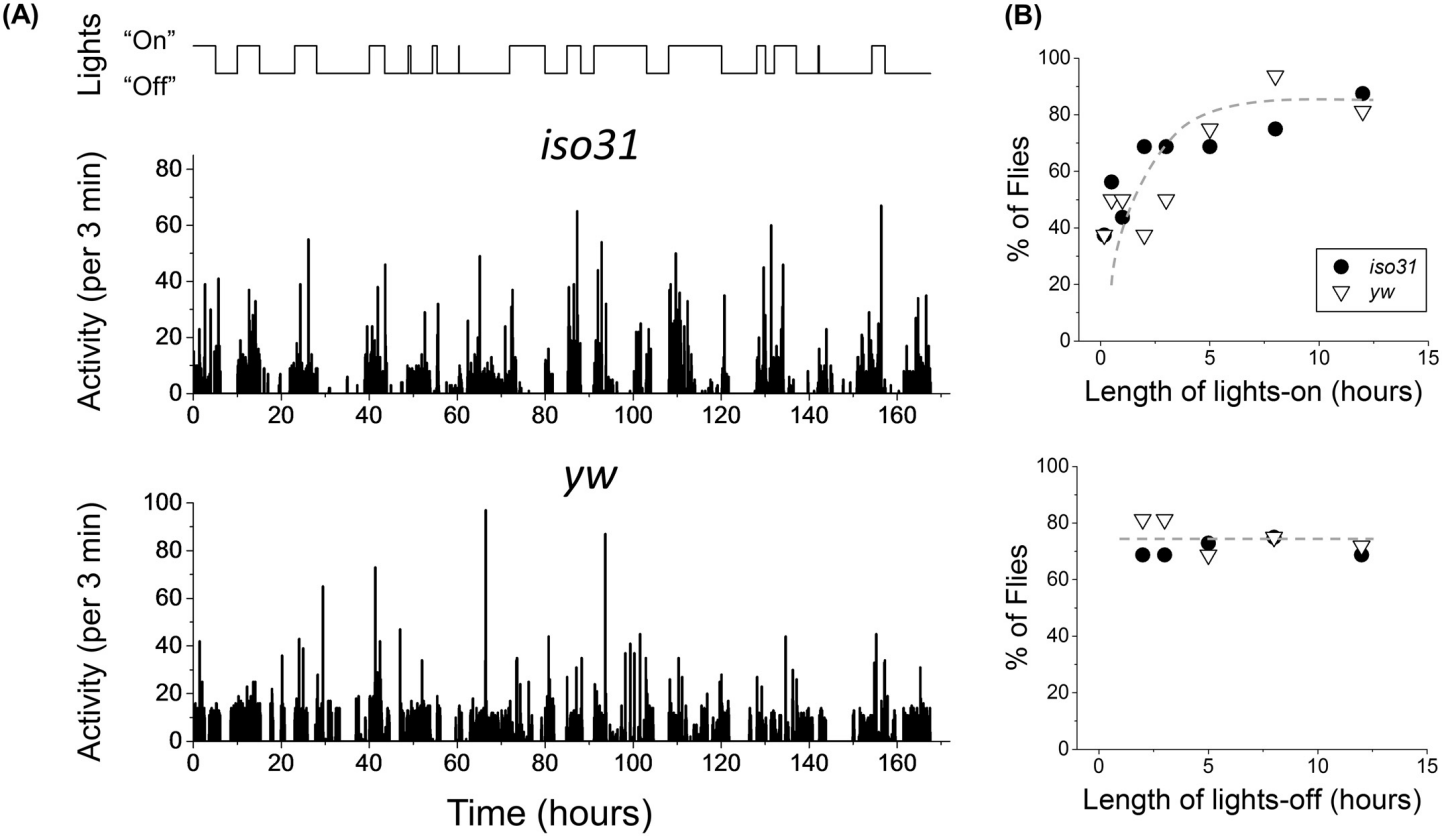
Experiments with light pulses show correlation between duration of “day” and occurrence of the burst. (A) Light pattern with various durations of day and night (top) and examples of activity profiles for *iso31* (middle) and *yw* (bottom). (B) The percentage of flies, which show the burst increases monotonically with increasing length of day (top), but remains roughly constant with changes in length of night (bottom). *iso31* shown in white triangles and *yw* in black circles, grey dashed curve is a guide to the eye, top and bottom panels.

In order to determine if the burst frequency depends on the length of illumination, we next placed flies under LD cycles of random length between 1–12 hours with the maximum light level at 8000 lux (Fig 5A, top). Since the burst can appear up to 5 hours after lights turn on, we ensured that a minimum of 5 hours of darkness was allowed following the 1–2 hour light pulses. Based on the transient nature of the burst, we naively hypothesized that an 8000 lux strong pulse for ~ 1 hour might be sufficient to elicit the behavior in majority of flies. Instead, we find ~40% of *yw* and *iso31* flies show the burst after a 1 hour pulse (Fig 5A and 5B, top). The rate of incidence increases for longer pulses and reach maximum at ~85% for ≥8 hours of illumination (Fig 5B, top). The data also show frequency of the behavior is independent of length of the lights-off period, suggesting that its incidence in the fly population was not constrained by insufficient interval between light pulses (Fig 5B, bottom). These results provide additional support for light being an essential trigger of the burst.

## Discussion

The M, E and, more recently, A peaks have received much attention in studies of fruit fly locomotion, with characterization of these peaks leading to a clearer description of fly activity on the circadian timescale [8,13,15,32]. In this paper we introduce a previously overlooked feature in fly activity that, unlike the M, E, and A peaks, occurs stochastically in time and consequently does not appear in ensemble-averaged data. In single fly recordings the feature appears as a transient yet pronounced burst in activity, lasting for ~3 min and showing activity on average ~10 standard deviations larger than basal levels.

Using a custom-built illumination system, we demonstrate that the burst in activity is strongly light dependent, with ~25% of flies showing the burst for 50 lux and rising to ~85% for 8000 lux in 12:12 LD cycles (Fig 3C). Consistent with this finding, our experiments under variable-length LD cycles indicate that a minimum of ~2 hour long pulse of 8000 lux is required to elicit the burst in at least 50% of flies (Fig 5B) and under DD conditions the burst is undetectable (data not shown). Similarly, mutation in the *histidine decarboxylase* gene that disrupts phototransduction through the eyes, deletion of *ninaE* that is required for expression of the Rh1 rhodopsin in R1-6 photoreceptors and deletion of *norpA* that eliminates overall photoreceptor potential—all significantly reduce detection rate of the burst (Fig 4H). Together these data suggest that the circuit underlying the burst is light-sensitive. Light-sensitivity in flies has been studied in behavioral contexts such as circadian rhythms and phototaxis. Light-sensitivity of the circadian clock comes primarily through the photopigment CRY that, when activated by blue light, can bind and degrade clock protein TIM in pacemaker neurons [33,34]. In our work *cry* ^*01*^ flies show burst at wildtype levels, indicating that the photopigment is an unlikely candidate in transducing light stimulus to the necessary circuit. Instead, photic pathway to the burst circuit is likely similar to that for *Drosophila* phototaxis, in which the R1-8 photoreceptors provide the first layer of light-sensitivity [35]. Consistent with this conjecture, the burst is strongly suppressed in *ninaE* and *norpA*. *hdc* mutants also show similar results, paralleling phototactic defects seen in animals with mutation in a histamine-gated channel HisCl1 [36].Lastly, results from the vision mutants also permit us to dissociate the burst from basal activity. For instance, *ninaE* and *norpA* display wild-type basal activity rates (Fig 4G, black and pink lines) while *hdc* animals are on average hyperactive (Fig 4G, peak on purple distribution shifted relative to others), yet all three display significantly reduced burst frequencies (Fig 4H). These results suggest that reduction in burst frequency is related to reduced phototransduction and is not a consequence of decrease in baseline activity.

While the frequency of the burst is highly dependent on the effective level of illumination, its timing and magnitude are not. Our data show that regardless of light levels the most likely time for the burst to appear is ~2.3 hours after lights turn on, show ~5 times more activity than the M peak (Fig 3D and 3E) and approximately 10 standard deviations away from baseline activity (see Methods). These attributes of the burst appear robust against differences in genetic background, a noteworthy distinction when contrasted against the well-studied M, E and A peaks, whose characteristics differ considerably between the tested *yw*, *iso31* and 2U lines (Fig 2 and S2 Fig). The robustness of the burst characteristics against genetic background is surprising, given fundamental behaviors such as sleep and nociception can vary considerably among standard wildtype lines [37,38]. Examination of additional wildtype strains will reveal fully the extent of this intriguing robustness.

Temporal stochasticity of the burst suggests the underlying circuit, contrary to the clock circuit, is likely susceptible to internal electrical and biochemical fluctuations (compare Fig 3D to S6A Fig). Indeed, its apparent independence from the circadian clock is consistent with the burst appearing at variable times and in animals that are behaviorally arrhythmic (Figs 3D and 4). Although the *per* ^*0*^ and *dbt* ^*AR*^ data indicate that active forms of PERIOD or DOUBLETIME are unlikely to be required for the burst, we cannot preclude possible roles by other clock genes, such as *timeless* and *clock*, which have been implicated in several non-clock functions [39,40]. Thus, although a functional clock does not seem to be necessary for the bursts, components of the clock machinery could still be involved. Returning to the topic of temporal regulation, stochasticity in behavior has been reported, for example, in saccades during *Drosophila* flight and in larval thermotaxis [41,42]. More broadly, variability in behavior often arises from intrinsically noisy or sparse neural pathways. Although it can limit some aspects of performance, such stochasticity can also enhance overall efficiency and creativity in behavior [43,44]. The variability in the burst timing particularly in the presence of bright light is reminiscent of a sparse circuit in which a limited number of components dictate the final output. Why the burst is expressed stochastically is currently unknown but will likely become clear once the actual nature of activity during the behavior is identified.

Despite its temporal stochasticity, the burst is robustly reproducible in a variety of flies. The robustness supports our hypothesis that the burst represents an important part of fly daily activity belonging to the rich repertoire of behaviors exhibited by the animal. Though our present studies cannot identify the nature of activity during the burst, here we briefly speculate on some possibilities. Given its short duration of ≲ 3 min, the burst is unlikely to constitute excursions along the entire length of the tube. Instead, it likely involves a repetitive action such as grooming at or near the IR beam [45]. Brief but rapid body or wing movements typical in grooming could produce the large activity counts seen in the bursts. Activity monitors with single IR beam have the drawback that they can detect fly movement only at one location. This raises the possibility that flies might perform the repetitive action at other locations along the tube as well, but interestingly, with the IR beam as a preferred location within the first few hours after lights turn on. The IR beam used in our experiments could also provide part of the explanation for the burst by implicating temperature sensation in the fly. Temperature measurements at the beam show a small < 1°C increase due to IR. This small difference in temperature may be sufficient to evoke a thermotactic response [46]. Indeed, our preliminary video recording of fly locomotion suggests that presence of the IR beam may increase frequency of the burst in the presence of light (data not shown). These findings would be consistent with several lines of evidence that have reported substantial overlap between molecules that regulate light and temperature circuits in *Drosophila* [46–48]. Since the burst appears more frequently at higher light intensities, there is a possibility that the burst is a stress or escape response to light. However, one would expect such a reaction to appear uniformly throughout the day and not be associated temporally with turning on of lights, as seen in our data.

In summary, we present here photoactivated transient bursts of activity seen in individual fruit fly locomotion. The burst appears stochastically, deviating from the prevailing pattern in fly locomotion that is currently dominated by rhythmic morning and evening peaks in activity. The robust yet stochastic nature of this behavior highlights a need for close examination of single fly recordings using flexible measurement systems such as the one introduced here. We foresee such measurements enriching our understanding of *Drosophila* daily activity in ways that population-level analyses with standard light patterns cannot.

## Supporting Information

S1 Fig The interior of the light control box and the remote. Main components and part numbers are labeled. A more detailed diagram of all parts of the light control system is available upon request.
(TIF)

S2 Fig Locomotor activity of control fly strains. Under LED-controlled light-dark conditions wild-type flies strains (iso31, yw and 2U) show similar behavior with morning and evening peaks, while in constant darkness their behaviors are markedly different. (A) Activity (top), actogram (middle) and power spectrum (bottom) of average locomotor activity of 16 iso31 flies for 3 days in LD. iso31 and yw show similar behavior. (B-D) Actogram (top) and power spectrum (bottom) of average locomotor activity of 16 2U (B), yw (C) and iso31 (D) flies for 14 days in DD. Black solid line shows power spectrum of first 7 days of DD recordings and grey dashed line of last 7 days. For LD activity data (A top) day/night are shown in white/black bars, “M” stands for morning peak and “E” for evening peak. (A-D) Data binned in 30 minutes. White/grey background represents actual day/night in LD and subjective day/night in DD.
(TIF)

S3 Fig Appearance of bursts during the light-dark cycle. (A) Examples of delay, Δt, between the burst and the moment light turns on for 32 flies measured for 7 days. First group of 16 flies are iso31 and the remaining 16 are yw. White and black bar on the right shows light/dark conditions at Δt. (B) Distribution of Δt calculated for 159 flies. Bursts are clustered into two groups, one centered ~2 hours and another ~10 hours. Standard deviation for the first group σ1 = 1.3 hours, and for the second σ2 = 2.7 hours.
(TIF)

S4 Fig Individual flies show significant burst of activity after start of morning activity. (A) Examples of activity for four different flies with maximum day light intensity of 100, 1000, 4000 and 8000 lux. Morning/evening peaks denoted as “M” and “E” respectively, burst of activity as “B”, Δt shows delay of B peak after light turns on. Activity shown with black columns; light patterns with black line. (B) Normalized autocorrelation functions for the burst (top left), the M peak (top right), random data (bottom left) and ideal single peak oscillation (bottom right).
(TIF)

S5 Fig Averaging or binning of data leads to masking of the burst. In the averaged locomotion data of 2 flies, bursts are much higher than the M peak. Consecutive averaging of multiple flies from 2 to 16 flies (A-C) results in the dominance of M and E peaks over the burst. Increasing the bin size (D) also conceals the burst in the actogram. (A-D) Activity shown in grey columns; light patterns in black line.
(TIF)

S6 Fig Timing of the M and E peaks in trapezoidal experiment. (A) Average time delay of the M peak after lights turn on, for different maximum daylight intensity. (B) Average advance timing of the E peak before light turns off. Negative values mean that peak occurs after light turns off. (C) Actual light intensity at the moment of the M peak. Legend and number of flies, yw (N = 32) and iso31 (N = 32) in (A) applies to panels (A-C).
(TIF)
