# Supplementary figures and images for: A Stochastic Burst Follows the Periodic Morning Peak in Individual Drosophila Locomotion
Source: PLoS One. 2015 Nov 3;10(11):e0140481. doi: 10.1371/journal.pone.0140481 (PMC4631454; doi:10.1371/journal.pone.0140481)

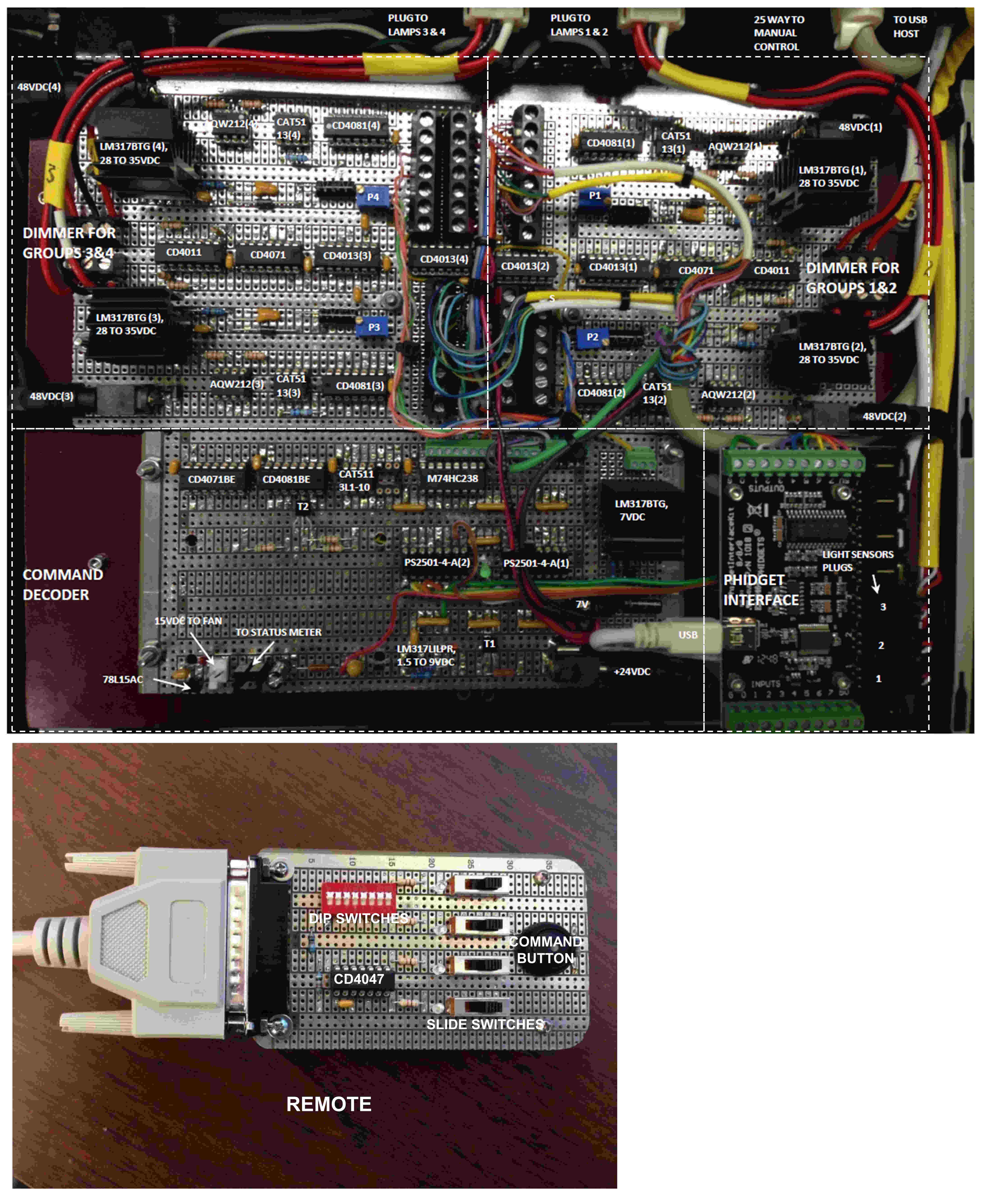

Supplement: S1 Fig — Main components and part numbers are labeled. A more detailed diagram of all parts of the light control system is available upon request. (TIF) [file pone.0140481.s001.tif]

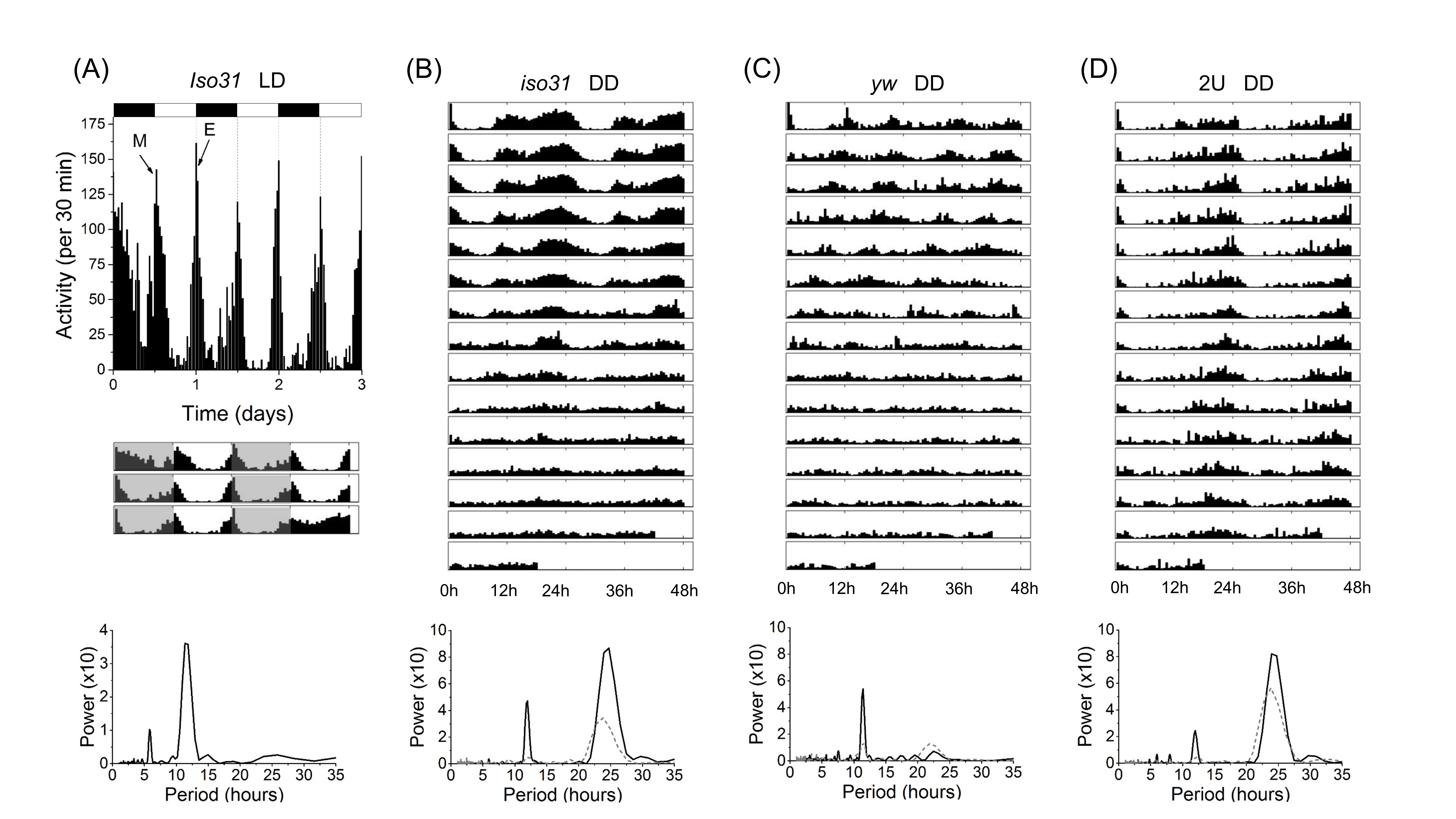

Supplement: S2 Fig — Under LED-controlled light-dark conditions wild-type flies strains (iso31, yw and 2U) show similar behavior with morning and evening peaks, while in constant darkness their behaviors are markedly different. (A) Activity (top), actogram (middle) and power spectrum (bottom) of average locomotor activity of 16 iso31 flies for 3 days in LD. iso31 and yw show similar behavior. (B-D) Actogram (top) and power spectrum (bottom) of average locomotor activity of 16 2U (B), yw (C) and iso31 (D) flies for 14 days in DD. Black solid line shows power spectrum of first 7 days of DD recordings and grey dashed line of last 7 days. For LD activity data (A top) day/night are shown in white/black bars, “M” stands for morning peak and “E” for evening peak. (A-D) Data binned in 30 minutes. White/grey background represents actual day/night in LD and subjective day/night in DD. (TIF) [file pone.0140481.s002.tif]

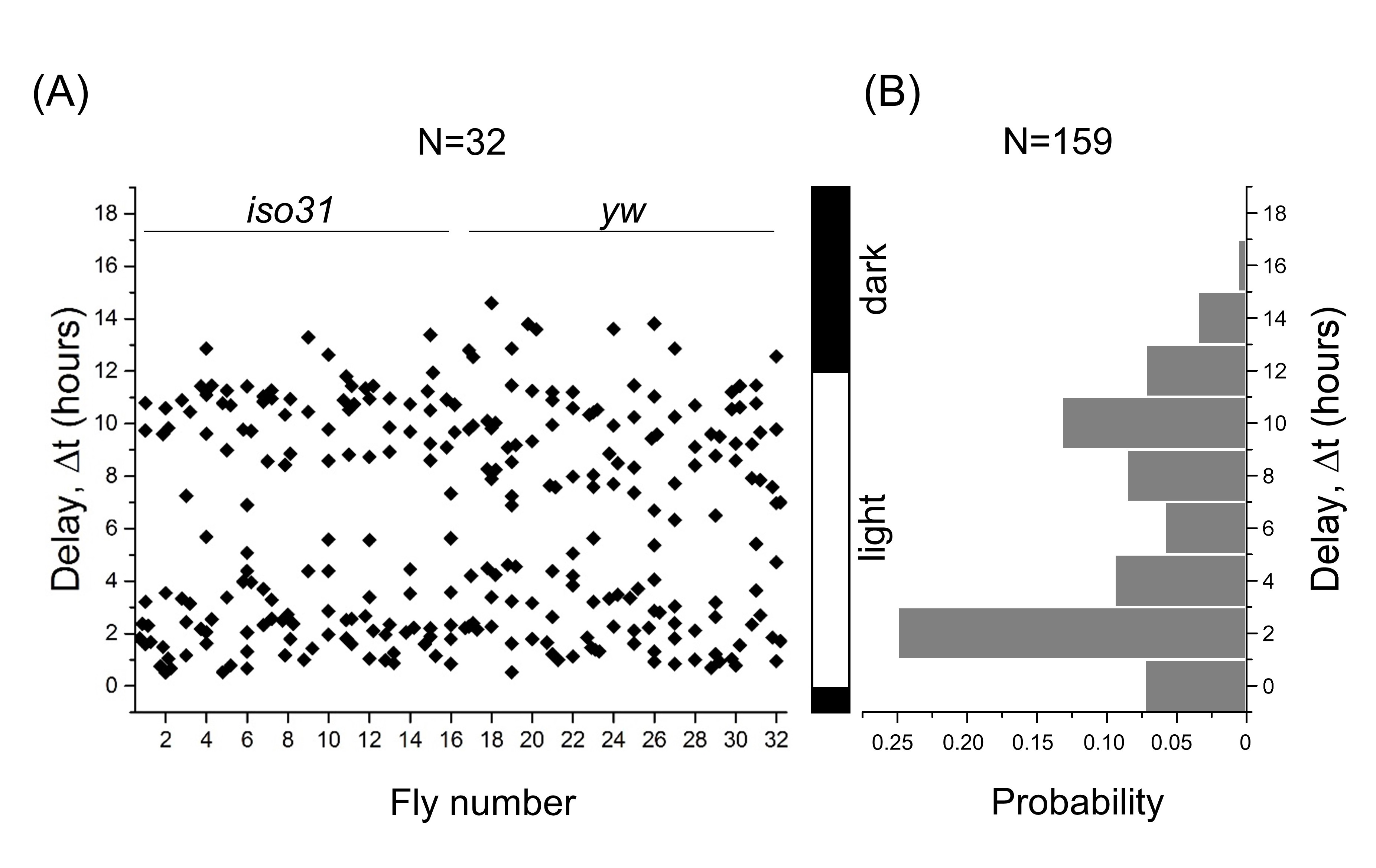

Supplement: S3 Fig — (A) Examples of delay, Δt, between the burst and the moment light turns on for 32 flies measured for 7 days. First group of 16 flies are iso31 and the remaining 16 are yw. White and black bar on the right shows light/dark conditions at Δt. (B) Distribution of Δt calculated for 159 flies. Bursts are clustered into two groups, one centered ~2 hours and another ~10 hours. Standard deviation for the first group σ1 = 1.3 hours, and for the second σ2 = 2.7 hours. (TIF) [file pone.0140481.s003.tif]

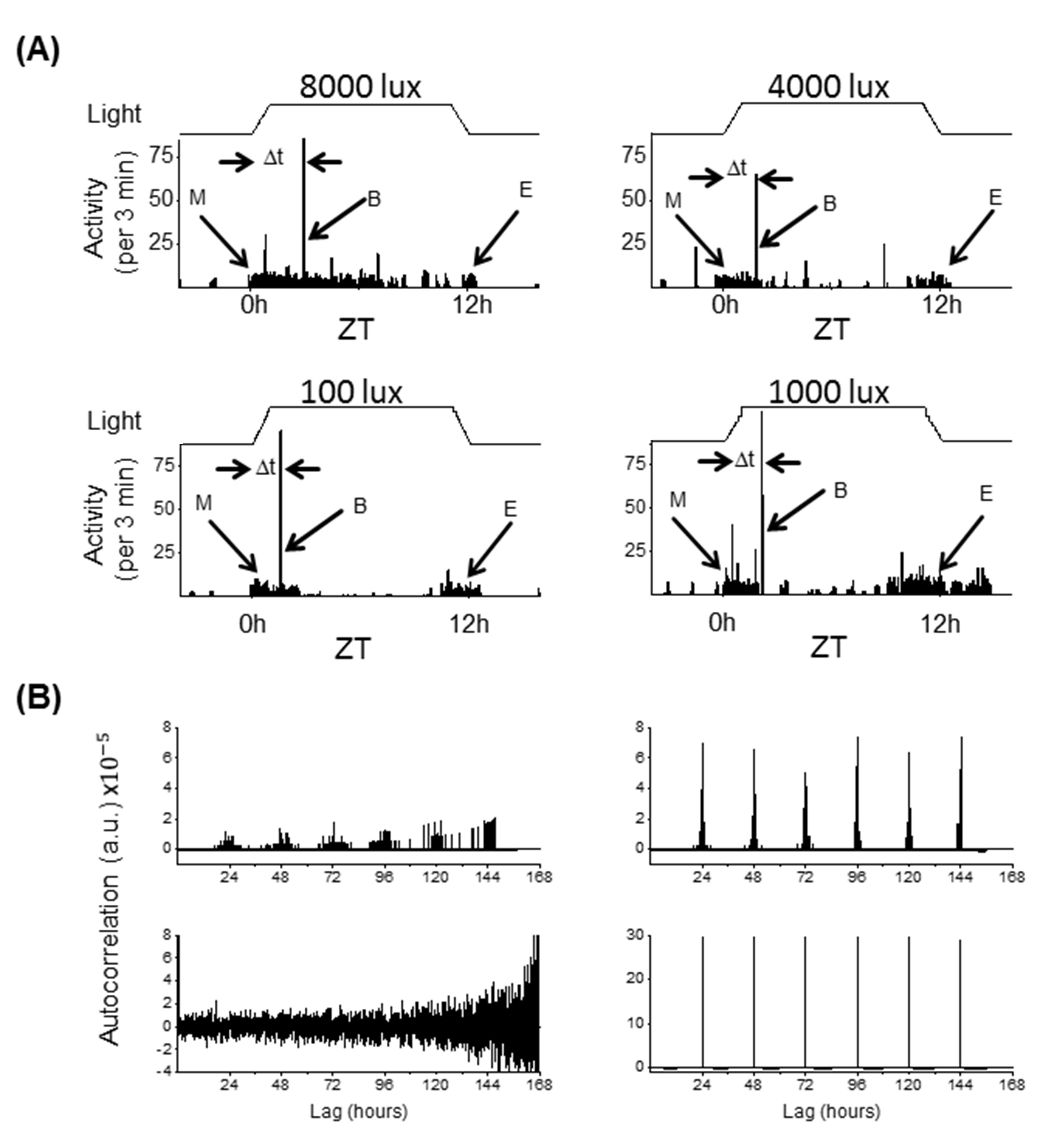

Supplement: S4 Fig — (A) Examples of activity for four different flies with maximum day light intensity of 100, 1000, 4000 and 8000 lux. Morning/evening peaks denoted as “M” and “E” respectively, burst of activity as “B”, Δt shows delay of B peak after light turns on. Activity shown with black columns; light patterns with black line. (B) Normalized autocorrelation functions for the burst (top left), the M peak (top right), random data (bottom left) and ideal single peak oscillation (bottom right). (TIF) [file pone.0140481.s004.tif]

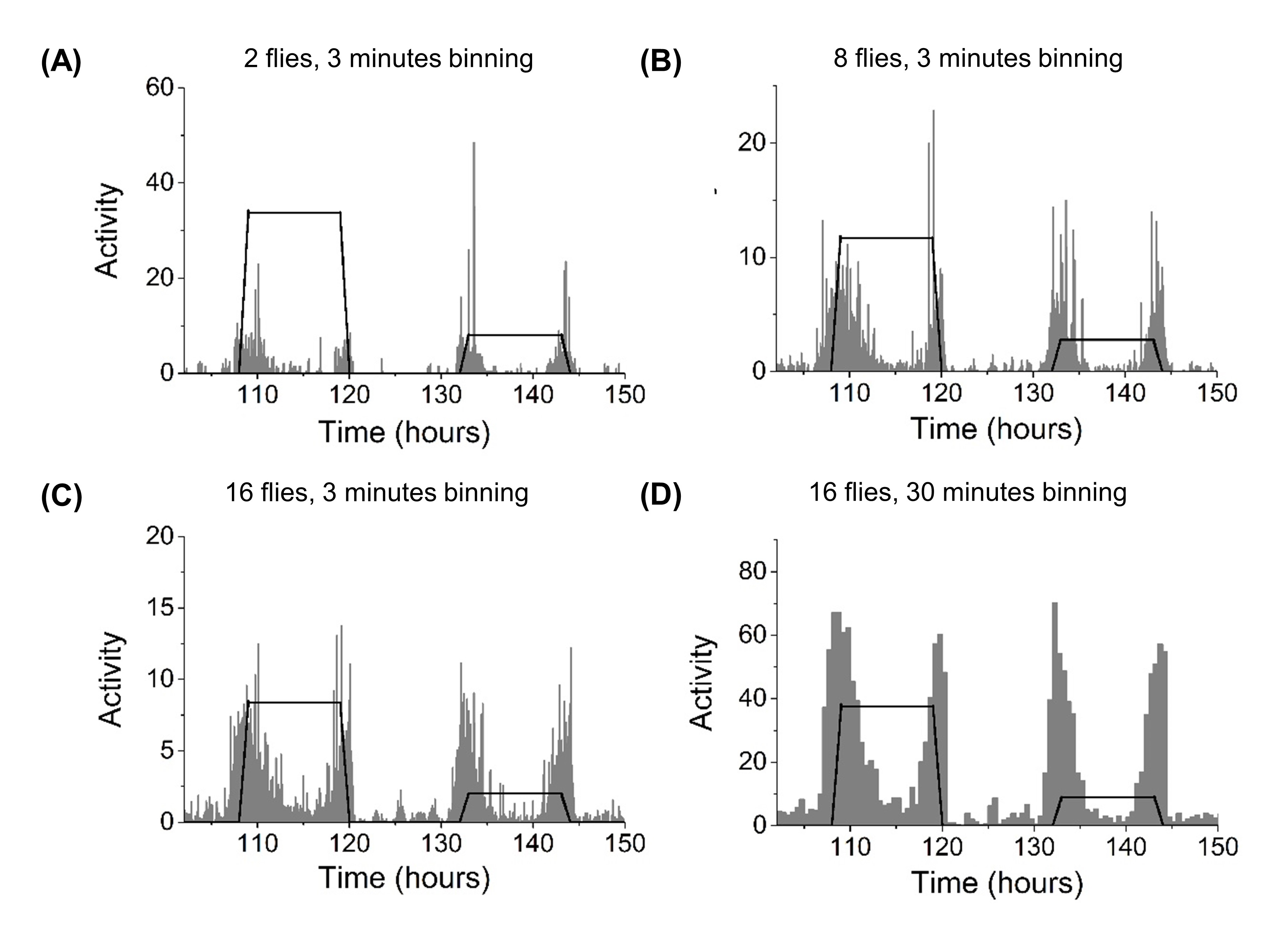

Supplement: S5 Fig — In the averaged locomotion data of 2 flies, bursts are much higher than the M peak. Consecutive averaging of multiple flies from 2 to 16 flies (A-C) results in the dominance of M and E peaks over the burst. Increasing the bin size (D) also conceals the burst in the actogram. (A-D) Activity shown in grey columns; light patterns in black line. (TIF) [file pone.0140481.s005.tif]

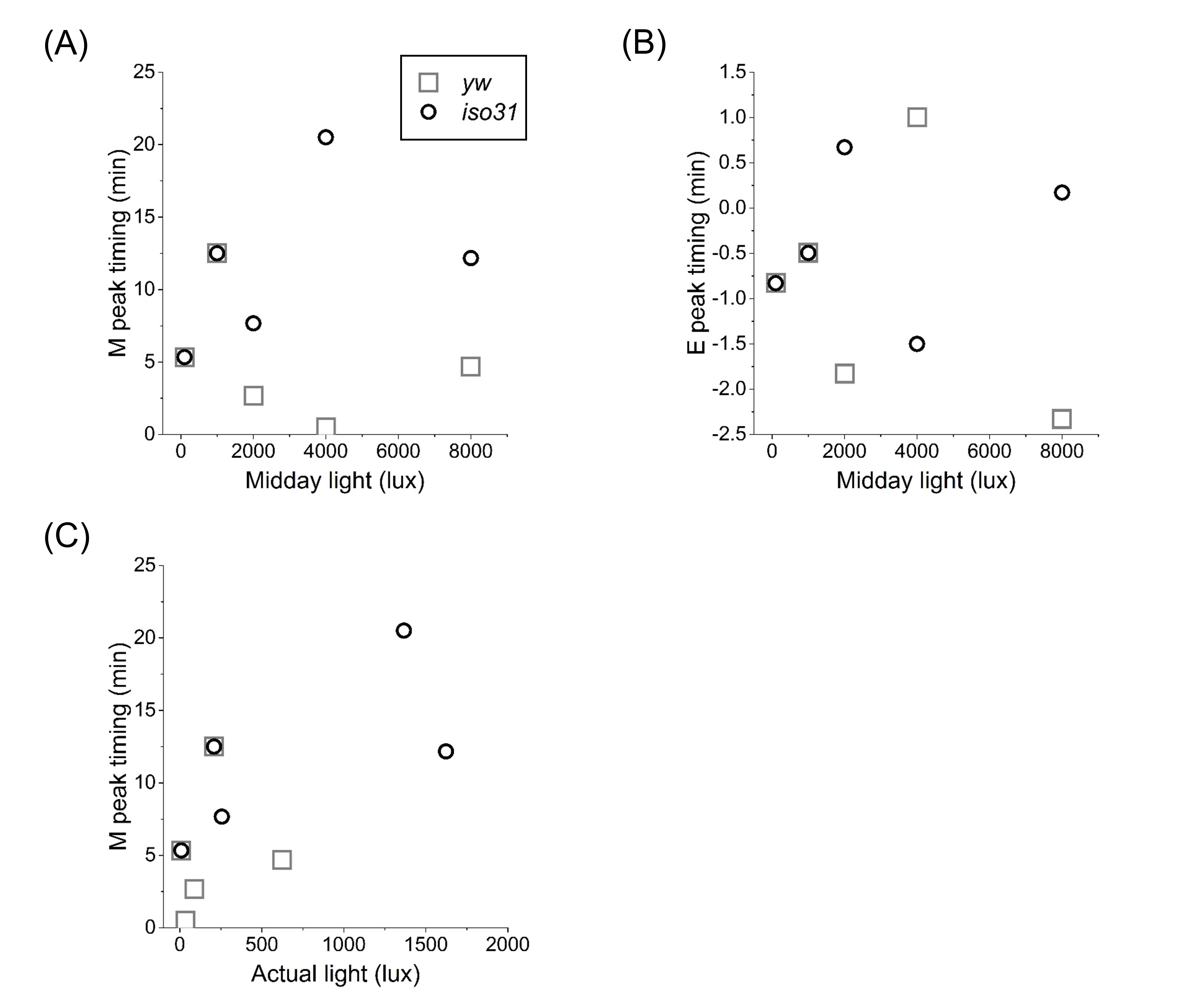

Supplement: S6 Fig — (A) Average time delay of the M peak after lights turn on, for different maximum daylight intensity. (B) Average advance timing of the E peak before light turns off. Negative values mean that peak occurs after light turns off. (C) Actual light intensity at the moment of the M peak. Legend and number of flies, yw (N = 32) and iso31 (N = 32) in (A) applies to panels (A-C). (TIF) [file pone.0140481.s006.tif]
